# Identification of Novel Inhibitors of DLK Palmitoylation and Signaling by High Content Screening

**DOI:** 10.1038/s41598-019-39968-8

**Published:** 2019-03-06

**Authors:** Dale D. O. Martin, Prasad S. Kanuparthi, Sabrina M. Holland, Shaun S. Sanders, Hey-Kyeong Jeong, Margret B. Einarson, Marlene A. Jacobson, Gareth M. Thomas

**Affiliations:** 1 Shriners Hospitals Pediatric Research Center, Lewis Katz School of Medicine at Temple University, 3500 N. Broad Street, Philadelphia, PA 19140 USA; 2 Fox Chase Cancer Center, Philadelphia, PA USA; 3 Moulder Center for Drug Discovery Research, Temple University School of Pharmacy, Philadelphia, USA; 4 Department of Anatomy and Cell Biology, Lewis Katz School of Medicine at Temple University, Philadelphia, USA

## Abstract

After axonal insult and injury, Dual leucine-zipper kinase (DLK) conveys retrograde pro-degenerative signals to neuronal cell bodies *via* its downstream target c-Jun N-terminal kinase (JNK). We recently reported that such signals critically require modification of DLK by the fatty acid palmitate, *via* a process called palmitoylation. Compounds that inhibit DLK palmitoylation could thus reduce neurodegeneration, but identifying such inhibitors requires a suitable assay. Here we report that DLK subcellular localization in non-neuronal cells is highly palmitoylation-dependent and can thus serve as a proxy readout to identify inhibitors of DLK palmitoylation by High Content Screening (HCS). We optimized an HCS assay based on this readout, which showed highly robust performance in a 96-well format. Using this assay we screened a library of 1200 FDA-approved compounds and found that ketoconazole, the compound that most dramatically affected DLK localization in our primary screen, dose-dependently inhibited DLK palmitoylation in follow-up biochemical assays. Moreover, ketoconazole significantly blunted phosphorylation of c-Jun in primary sensory neurons subjected to trophic deprivation, a well known model of DLK-dependent pro-degenerative signaling. Our HCS platform is thus capable of identifying novel inhibitors of DLK palmitoylation and signalling that may have considerable therapeutic potential.

## Introduction

In both chronic neuropathological conditions and following acute injury, Dual Leucine-zipper Kinase (DLK) signals *via* its downstream target c-Jun N-terminal Kinase (JNK) to activate pro-degenerative transcription and subsequent neuronal death^1–7^. Genetic knockout of DLK confers striking neuroprotection in several models of neurodegeneration, spurring great interest in targeting DLK therapeutically as a neuroprotective strategy^1,2,5,7^. Indeed, inhibitors of DLK’s kinase activity have shown therapeutic promise in multiple animal models of disease^1,8–10^. Unfortunately, though, the most promising DLK inhibitors reported thus far also inhibit additional kinases^8^, which may limit the potential of this therapeutic approach.

An alternative or complementary strategy that holds considerable promise would be to target DLK-specific regulatory features. Our studies of DLK-specific regulation led to our recent finding that DLK undergoes palmitoylation^11^, the reversible covalent addition of a saturated fatty acid, typically palmitate^12–14^. Palmitoylation is best known to control protein subcellular localization and we found that palmitoylation targets DLK to specific axonal vesicles in primary sensory neurons^11^. ‘Hitch-hiking’ on these vesicles may allow DLK to convey retrograde signals from damaged axons to neuronal cell bodies^11^. Interestingly, though, palmitoylation plays an unexpected additional role, because it is also critical for DLK to phosphorylate and activate ‘downstream’ JNK pathway kinases^11^. Consistent with the importance of palmitoylation for DLK-JNK signaling, genetically mutating DLK’s palmitoylation site prevented JNK phosphorylation in non-neuronal cells, and blocked JNK-dependent responses to axonal injury in cultured neurons^11^. These findings suggested to us that compounds that prevent DLK palmitoylation might be as neuroprotective as inhibitors of DLK’s kinase activity. However, pursuing this therapeutic strategy would require development of an effective screening method to identify such compounds.

Here we report that in non-neuronal cells, DLK localization is also highly palmitoylation-dependent. This localization can be used as a proxy for DLK palmitoylation that is compatible with a High Content Screening (HCS) approach. We optimized our screen to identify and eliminate compounds that broadly affect protein transcription, translation and/or stability and to eliminate likely cytotoxic compounds. Using these optimized conditions we screened a library of FDA-approved compounds and identified several that specifically affect DLK localization. Ketoconazole, an antifungal agent that most dramatically affected DLK localization in our primary screen, also inhibited DLK palmitoylation in follow-up biochemical assays and reduced DLK-dependent signaling in primary neurons. Our screening assay thus has the potential to identify novel modulators of DLK palmitoylation, which may have considerable therapeutic potential.

## Results

### DLK subcellular localization is highly palmitoylation-dependent in HEK293T cells

**Figure 1:**
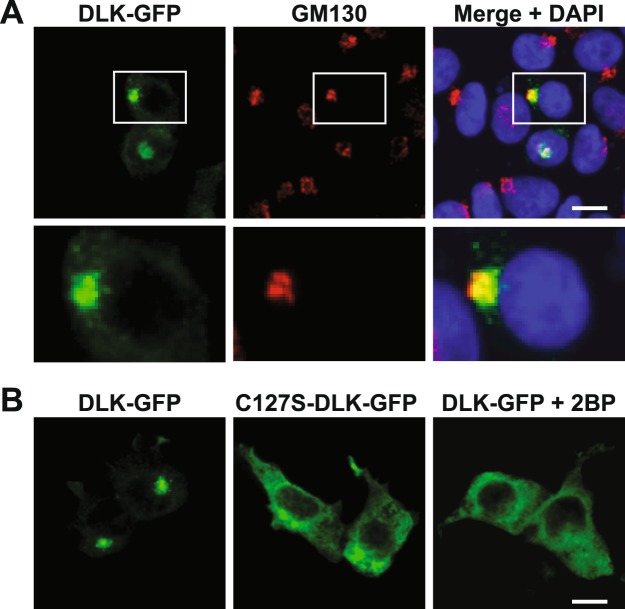
Palmitoylation-dependent localization of DLK-GFP to the Golgi apparatus in HEK293T cells. (**A**) HEK293T cells were transfected to express wild type DLK-GFP and subsequently fixed. DLK-GFP and the Golgi marker GM130 were detected with specific antibodies and nuclei were detected using the DNA dye DAPI. (**B**) HEK293T cells were transfected as in *A* to express either wild type DLK-GFP (DLK-GFP) or a DLK palmitoylation site mutant (C127S-DLK-GFP). C127S mutation, or treatment with the palmitoylation inhibitor 2BP, diffuses the Golgi-associated clusters of DLK-GFP.

In primary sensory neurons, DLK localizes to axonal vesicles^11^. This discrete localization is prevented by a pharmacological inhibitor of protein palmitoylation (the compound 2-Bromopalmitate (2BP^15^)) or by point mutation of DLK’s palmitoylation site, Cys-127^11^. Subcellular localization changes of this type are often used as readouts in High Content Screening (HCS)^16,17^, an approach that might hence be well suited to identify compounds that inhibit DLK palmitoylation. However, because a non-neuronal cell line might be more amenable to HCS approaches than primary neurons, we assessed whether DLK localization is also palmitoylation-dependent in HEK293T cells. We found that transfected wild type GFP-tagged DLK (wtDLK-GFP) in HEK293T cells localizes to intracellular membranes that colocalize with the Golgi marker GM130 (Fig. 1A). wtDLK’s Golgi localization in HEK293T cells may be because the axonal vesicle population is not present in this cell line and/or because many mammalian palmitoyl acyltransferases (PATs, which catalyze palmitoylation) localize to the Golgi in these cells^18^. Importantly, though, this localization was again highly palmitoylation-dependent, because both 2BP treatment and C127S mutation shifted DLK localization from Golgi-associated to diffuse (Fig. 1B).

### Palmitoylation-dependent control of DLK localization is a robust, HCS-compatible readout

Given that 2BP treatment and C127S mutation completely eliminate DLK palmitoylation in biochemical assays^11^, Golgi localization of DLK can thus serve as an effective proxy for DLK palmitoylation that may be HCS-compatible. To test this possibility, we seeded HEK293T cells in 96 well plates, prior to transfection with wtDLK-GFP and subsequent treatment with 2BP (positive control) or vehicle. We then fixed cells and acquired images of wtDLK-GFP localization using an ImageXpress High Content Analyzer. After thresholding each image to an identical value, we used HCS software to assess six different metrics of wtDLK-GFP’s punctate distribution (total puncta count, puncta per cell, puncta area per cell, total puncta area, puncta integrated intensity and puncta average intensity). Values presented ‘per cell’ were normalized to the total number of cells per field, measured using the nuclear marker DAPI. 2BP significantly decreased each of these readouts of DLK punctate distribution (Supplementary Fig. 1). We also assessed the robustness of each of these readouts by calculating the z-prime (z′), a statistical measurement commonly used to evaluate and validate HCS assays^19^. Typically, a z′ value of ≥0.5 is deemed an excellent assay. Five of the six readouts yielded a z′ value of approximately 0.5 (Supplementary Fig. 1). The wtDLK-GFP localization change is therefore an HCS-compatible proxy readout of DLK palmitoylation. Of the six readouts we chose to focus on DLK-GFP ‘puncta per cell’, because we surmised that the ability to normalize effects to number of cells per well could be important in ‘downstream’ validation assays.

### An optimized high-throughput imaging screen for DLK palmitoylation

**Figure 2:**
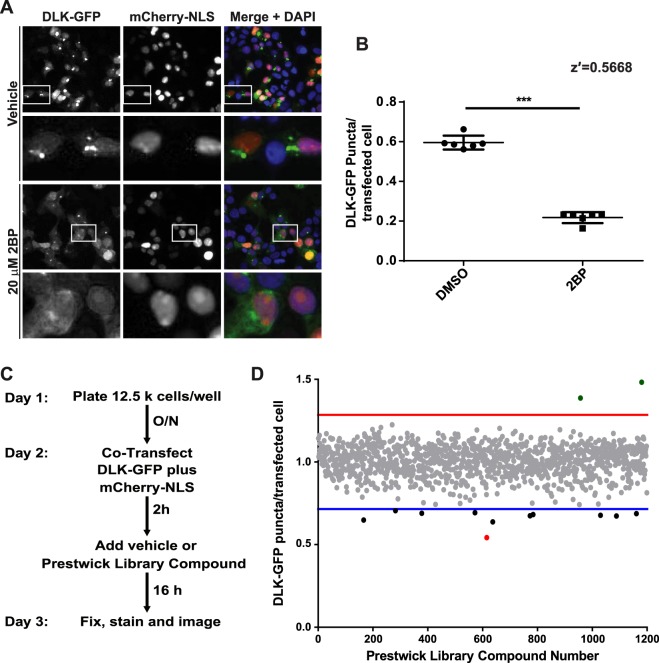
A High Content Imaging screen identifies ketoconazole as the most potent compound to inhibit DLK-GFP puncta formation. (**A**) HEK293T cells cotransfected with DLK-GFP plus mCherry-NLS were treated with 2BP or vehicle and fixed to detect GFP, mCherry and the nuclear marker DAPI. 2BP reduces DLK-GFP puncta without affecting mCherry-NLS expression or DAPI signal. Scale bar: 25 µm. (**B**) HEK293T cells were seeded into 12 wells of a 96-well plate and transfected with DLK-GFP and NLS-mCh cDNA and then treated with 2BP (20 μM in DMSO) or 0.1% (v/v) DMSO vehicle as in *A*. Cells were fixed and imaged using an ImageXpress High Content Imaging system to detect GFP and NLS-mCh signals. Assay quality was determined by calculating the z-prime (z′) for 6 determinations per condition (z′ = S/R, S = [(Mean of Vehicle treated – 3xSD)-(Mean of 2BP – 3xSD)], R = Vehicle Mean – 2BP mean). (**C**) Design of the high-throughput screen for compounds that inhibit DLK-GFP punctate localization. (**D**) The effect of 1200 compounds from the Prestwick Chemical Library™ on DLK puncta per transfected cell (mean of 2 determinations per compound) was calculated using ImageXpress Image Analysis ‘TransFluor’ and Multi-Wavelength Scoring (MWS) modules. Compounds that decreased the number of transfected cells (from mCherry-NLS count) or the total number of cells (from DAPI count) by greater than 30%, relative to the mean of vehicle treated controls, are not plotted due to likely cytotoxicity or non-specific effects. Red and blue lines indicate 3 standard deviations (3 SD) above and below the mean of all determinations, respectively. Compounds that decreased DLK puncta per transfected cell below this 3 SD cut-off were considered ‘Hits’. The most potent ‘hit’, ketoconazole, is highlighted in red.

Our goal in establishing the HCS assay was to identify compounds that reduce DLK puncta number because they reduce DLK palmitoylation. However, we realized that DLK puncta numbers might also be reduced by compounds that impaired DLK transcription or translation, or by cytotoxicity. To facilitate detection of ‘false positive’ compounds that broadly affect these processes, we adapted our assay to incorporate a cotransfected cDNA that codes for a nuclear localization signal (NLS) fused to the red fluorescent reporter mCherry (mCherry-NLS)^20^, expressed downstream of the same CMV promoter used in the DLK-GFP cDNA. We continued to include the nuclear marker DAPI to quantify healthy nuclei per well because reduced DAPI counts and/or nuclear fragmentation (which are  detectable by ImageXpress software) could serve as an additional indicator of potentially cytotoxic compounds. Importantly, 2BP affected neither mCherry-NLS nor DAPI counts at the concentration used (Fig. 2A). In addition, the z′ value of our chosen metric (wtDLK-GFP puncta) improved when normalized to transfected cells per field (using mCherry-NLS count; Fig. 2B, z′ value of 0.567) rather than total cells per field (using DAPI count; Supplementary Fig. 1, z′ = 0.459).

### A Prestwick Chemical Library^TM^ screen reveals that the compound Ketoconazole reduces DLK punctate localization

Having validated our HCS approach with the positive control ‘tool compound’ 2BP, we sought to expand our assay to perform an initial library screen. The Prestwick Chemical Library™ consists of over 1200 compounds that have been approved by the FDA, EMA, or other agencies for use in humans^21^. The library was prepared by medicinal chemists and pharmacists to ensure high chemical diversity and known bioavailability in humans, thereby increasing the likelihood of identifying “high quality” hits. Another advantage of the Prestwick library is that positive hits have the potential to be used immediately in downstream analyses and studies.

Using our optimized conditions we therefore expanded our assay to screen the 1200 Prestwick library compounds (Fig. 2C). Compounds that reduced either mCherry-NLS or DAPI signals by more than 30%, relative to the mean of vehicle-treated controls, were excluded from further analysis due to likely cytotoxicity or effects on transcription/translation/protein stability. The effect of all remaining compounds on DLK puncta, relative to the total mCherry-NLS count (“DLK puncta per transfected cell”), was then quantified (Fig. 2D). Eleven compounds reduced DLK puncta per transfected cell by >3X SD, relative to the mean value for all compounds (Table 1). The antifungal compound ketoconazole had the greatest effect, reducing DLK puncta per transfected cell by 45.8 ± 0.5%, and was selected for follow-up studies.

**Table 1:** Compounds identified in the Prestwick Compound Library that reduced DLK-GFP puncta per transfected cell (“Puncta/NLS”) by > 3xSD, relative to the mean of all compounds tested.

Rank	Drug	Puncta per transfected cell (Read 1)	Puncta per transfected cell (Read 2)	Puncta per transfected cell (Mean)	Notes
1	Ketoconazole	0.537	0.547	0.542	Antifungal. Inhibits 17α-hydroxylase and 17,20-lyase activities of cytochrome P450 17A1, which are involved in steroid synthesis.
2	Lidoflazine	0.687	0.588	0.637	Ca^2+^ channel blocker. Coronary vasodilator.
3	Bromocryptine mesolate	0.665	0.642	0.649	Dopamine agonist. Also known as Parlodel.
4	Thioguanosine	0.694	0.652	0.673	Chemotherapeutic
5	Nefazodone HCl	0.688	0.661	0.674	5-HT2A receptor antagonist
6	Sulconazole nitrate	0.662	0.692	0.677	Antifungal. Also known as Exelderm.
7	Nifedipine	0.671	0.693	0.682	Ca^2+^ channel blocker. Also known as Adalat.
8	Tyloxapol	0.679	0.696	0.687	Non-ionic polymer. Component of the expectorant Tacholiquin.
9	Disulfiram	0.653	0.727	0.690	Inhibits acetaldehyde dehrogenase. Also known as Antubuse and Antabus
10	Imatinib	0.665	0.721	0.693	Inhibts bcr-abl for treatment of chronic myelogenous leukemia. Also known as Gleevec
11	Clomiphene citrate (ZE)	0.687	0.723	0.705	Selective estrogen receptor modulator (SERM)

Values for each of 2 replicates (Read 1, Read 2) are shown, together with the corresponding mean value.

### Ketoconazole inhibits DLK puncta formation and palmitoylation

**Figure 3:**
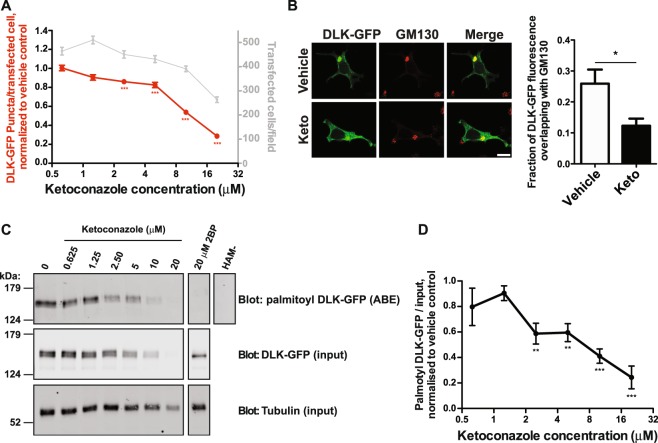
Dose-dependent inhibition of DLK-GFP localization and palmitoylation by ketoconazole. (**A**) Quantified DLK puncta per transfected cell from HEK293T cells transfected as in Fig. 2 and treated with the indicated concentrations of ketoconazole. Data (mean ± SEM) are plotted relative to DMSO vehicle control for 6 fields of view per condition from 8 determinations per condition. ***p < 0.001 compared to vehicle-treated control, ANOVA with *post hoc* Tukey test. F(6) = 14.6. Similar results were obtained in another experiment. (**B**) *Left panels* Images of HEK293T cells transfected with DLK-GFP cDNA and treated with 10 μM ketoconazole (‘Keto’) or vehicle, prior to fixation and immunostaining with anti-GFP and anti-GM130 antibodies. Scale bar: 10 μm. *Right panel* Quantified data from n = 22–24 cells per condition confirm that ketoconazole significantly reduces the fraction of DLK-GFP that colocalizes with GM130. *p < 0.05, t-test. (**C**) Palmitoyl-DLK levels (detected following Acyl-Biotin Exchange (ABE) assay to purify palmitoyl-proteins, top panel) from HEK293T cells transfected with DLK-GFP cDNA and subsequently treated with the indicated concentrations of ketoconazole. Middle and lower panels show total levels of DLK and tubulin, respectively, detected by western blotting of parent lysates. A negative control sample was generated by combining equal fractions of lysates from all conditions, which was then subjected to ABE in the absence of the key reagent hydroxylamine (HAM-). Molecular weight markers are indicated on each blot. (**D**) Quantified data from n = 3–7 determinations per condition from *C* confirm that ketoconazole significantly decreases palmitoyl:total DLK-GFP levels. **p < 0.01. ***p < 0.001 relative to vehicle treated cells, ANOVA with Bonferroni *post hoc* test, F(6) = 11.16. Some data from this panel are re-plotted in Fig. 4B.

We first assessed the dose-dependence of ketoconazole’s effect on DLK localization using a re-purchased stock of the compound. At the concentration used in the initial screen (10 µM) Ketoconazole again greatly reduced the number of DLK-GFP puncta/transfected cell (Fig. 3A) and also reduced the fraction of DLK-GFP fluorescence that colocalized with the Golgi marker GM130 (Fig. 3B). Ketoconazole’s effect on DLK-GFP puncta/transfected cell was clearly dose-dependent, first reaching statistical significance at 2.5 µM (Fig. 3A). At concentrations >10 µM, ketoconazole more markedly reduced the number of DLK-GFP puncta/transfected cell, but also clearly affected the number of transfected cells. However, these findings suggested that a clear window exists within which ketoconazole reduces DLK-GFP puncta/transfected cell without affecting overall transcription/translation/protein stability.

To determine whether effects of ketoconazole on DLK localization were linked to reduced palmitoylation, we subjected lysates of DLK-GFP-expressing cells to an orthogonal mechanism of action assay, Acyl-Biotin Exchange (ABE). In this assay, thioester-linked acyl modifications (i.e. palmitoylation) are exchanged for biotin and the resultant biotinyl-proteins are affinity-purified from cell lysates using avidin-conjugated beads^11,22^. Consistent with our findings from the DLK-GFP localization assay (Fig. 3A), ketoconazole dose-dependently decreased palmitoylation of DLK-GFP in ABE assays (Fig. 3C,D). At 2.5 µM, ketoconazole predominantly affected DLK palmitoylation, while at 10 µM, ketoconazole reduced DLK palmitoylation to a greater extent but also reduced total levels of DLK-GFP expression (Fig. 3C,D). At >10 µM, ketoconazole reduced tubulin levels, consistent with the reduced mCherry-NLS counts seen in the DLK-GFP localization assay, and again suggesting possible effects on transcription/translation and/or cytotoxicity. Perhaps surprisingly, though, even high concentrations of ketoconazole were not directly cytotoxic, because only a small percentage (<5%) of ketoconazole-treated cells stained positively for Propidium Iodide, a cell-impermeant dye that can be used to identify dead cells (Supplementary Fig. 3A). Consistent with this finding, only the highest concentration of ketoconazole (20 µM) significantly reduced the total number of cells per well (as identified by DAPI counts in the original HCS assay; Supplementary Fig. 3B). Results from our primary assay and follow-up immunocytochemical and biochemical assays thus suggest that there is therefore a window within which ketoconazole reduces DLK punctate localization and palmitoylation without broadly affecting protein transcription, translation or stability.

### Ketoconazole inhibits palmitoylation of DLK and PSD-95, but not GAP43

**Figure 4:**
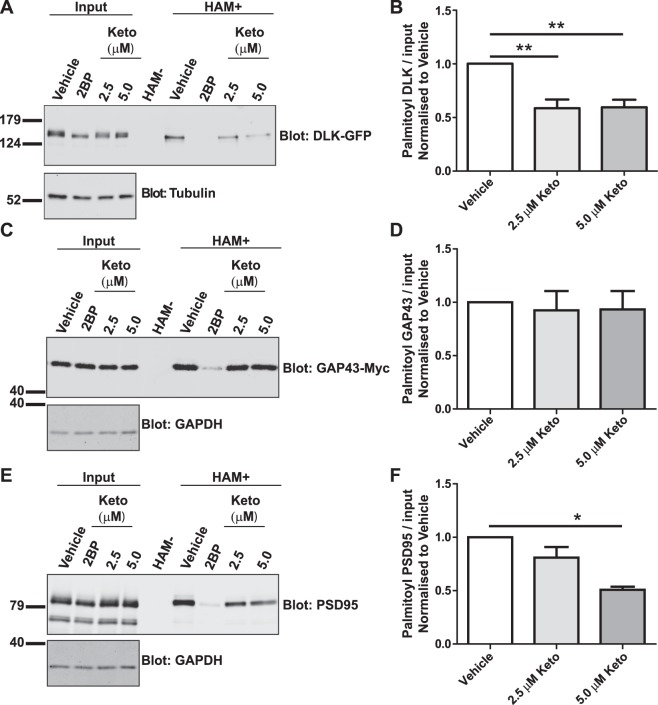
Ketoconazole inhibits palmitoylation of DLK and PSD-95, but not GAP43. (**A**) HEK293T cells were transfected with DLK-GFP cDNA and incubated with 20 µM 2BP, or with 2.5 µM or 5 µM ketoconazole 2 h post-transfection for 16–18 h. Upper western blot shows DLK total expression and palmitoyl-DLK levels (from ABE, ‘HAM+’) for each condition. A negative control sample was generated by combining equal fractions of lysates from all conditions, which was then subjected to ABE in the absence of the key reagent hydroxylamine (HAM-). Lower western blot shows tubulin levels, an indication of total protein expression. Molecular weight markers are indicated on each blot. Differing band widths in this panel are likely due to different protein concentrations and/or ionic strength of total lysates versus ABE fractions. (**B**) Histogram of pooled data (mean ± SEM) for 4 determinations per condition from *A*. Ketoconazole and 2BP both significantly reduce DLK palmitoylation. Some data from this panel are re-plotted as part of Fig. 3D. (**C**) As *A*, except that cells were transfected with GAP43-Myc cDNA ABE fractions were blotted with anti-Myc antibody and cell lysates were blotted to detect total expression of GAP43-myc (upper panel) and GAPDH (lower panel). (**D**) Histogram of pooled data (mean ± SEM) for 7 determinations per condition from *C*. Ketoconazole does not reduce GAP43 palmitoylation, but 2BP does. (**E**) As *C*, except that cells were transfected with PSD-95 cDNA and total lysates and ABE fractions were blotted with anti-PSD-95 antibody. (**F**) Histogram of pooled data (mean ± SEM) for 4 determinations per condition from *E*. 5 μM ketoconazole and 2BP both reduce PSD-95 palmitoylation. One-way ANOVA, Kruskal-Wallis post-hoc analysis; (**A**) ANOVA p = 0.0009, h = 13.92, (**C**) ANOVA not significant, (**E**) ANOVA p = 0.0158, h = 8.290.

We next sought to assess the extent to which effects of ketoconazole on cellular palmitoylation mirror those of the broad spectrum palmitoylation inhibitor 2BP. To address this question, we used ABE to assess palmitoylation of two other well characterized palmitoyl-proteins, Growth-Associated Protein-43 (GAP43) and Post-Synaptic Density-95 (PSD-95)^23,24^. In transfected HEK293T cells, ketoconazole significantly decreased palmitoylation of both DLK-GFP and PSD-95 (Fig. 4A,C), but did not reduce GAP43-Myc palmitoylation (Fig. 4B). In parallel assays, 2BP reduced palmitoylation of all three proteins. These findings suggest that ketoconazole is not a broad spectrum inhibitor of protein palmitoylation and is thus distinct from 2BP.

### Ketoconazole inhibits DLK-dependent cJun phosphorylation in sensory neurons subjected to trophic deprivation

**Figure 5:**
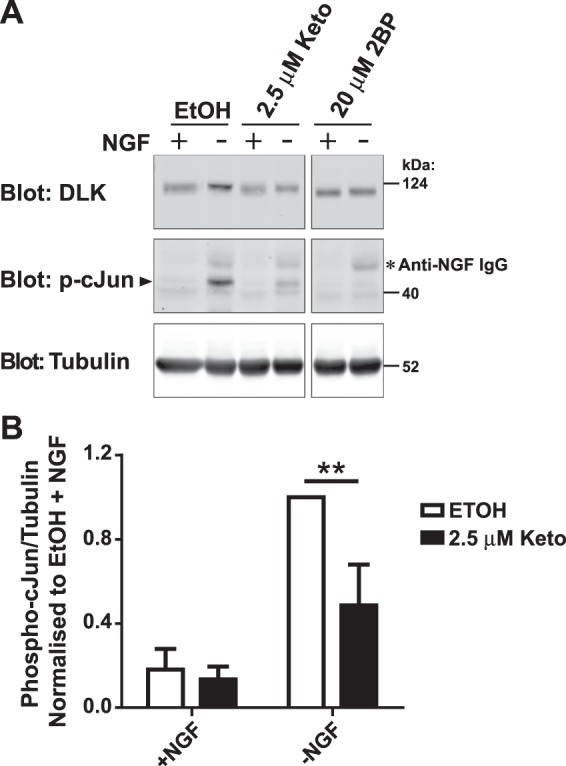
Ketoconazole significantly decreases DLK-mediated phospho-cJun activation in primary neurons. (**A**) Dorsal Root Ganglion (DRG) neurons were treated at 7 Days *in vitro* (DIV 7) with 2.5 µM Ketoconazole overnight or 20 µM 2BP for 2 h prior to a 2.5 h NGF withdrawal in presence of the indicated compound. Cells were lysed in SDS-PAGE loading buffer and levels of endogenous DLK, phospho-cJun (p-cJun) and tubulin were detected by western blot as indicated. The secondary antibody used on the p-cJun blot also weakly recognizes residual anti-NGF IgG used as part of the trophic factor deprivation (indicated by asterisk). (**B**) Quantification of phospho-cJun normalised to –NGF vehicle-treated cells. Two-way ANOVA indicates significant effects of interaction (p = 0.0071), NGF (p = 0.0026) and Ketoconazole (p = 0.0001). The effect of Ketoconazole in DRGs undergoing NGF withdrawal was also significant as determined by the Bonferroni post-test (p < 0.01). Error bars represent SEM.

Given the importance of palmitoylation for DLK-dependent signalling^11^ and the clear effects of ketoconazole on DLK palmitoylation levels (Figs 3C and 4A), we next assessed whether ketoconazole could affect neuronal DLK signalling. Dorsal Root Ganglion (DRG) sensory  neurons subjected to Trophic Deprivation (TD) activate a pro-degenerative DLK-JNK signaling pathway that leads to the phosphorylation of the transcription factor c-Jun^25,26^. Consistent with our prior finding that c-Jun phosphorylation requires palmitoyl-DLK^11^, 2BP completely prevented TD-induced c-Jun phosphorylation in DRG neurons (Fig. 5). Interestingly, ketoconazole also significantly reduced TD-induced c-Jun phosphorylation in sister cultures subjected to TD (Fig. 5). These findings suggest that ketoconazole can reduce not only DLK localization and palmitoylation, but also DLK-dependent neuronal signalling.

## Discussion

There is considerable interest in inhibiting DLK signaling as a therapeutic strategy to prevent neurodegeneration in a variety of pathological conditions. Although direct inhibitors of DLK’s kinase activity are being developed^1,8–10^, a complementary neuroprotective approach might be to prevent DLK palmitoylation, because this lipid modification is essential for DLK’s kinase activity^11^. Our high content imaging screen facilitates this latter approach by exploiting dramatic palmitoylation-dependent changes in DLK localization to identify compounds that inhibit DLK palmitoylation. Our screening method is robust and is capable of identifying compounds that reduce DLK palmitoylation in orthogonal biochemical assays and which also reduce DLK-dependent pro-degenerative signaling in neurons. Moreover, because inhibition of palmitoylation has not been pursued as a neuroprotective strategy, our screening platform has the potential to identify novel classes of compounds that may have considerable therapeutic potential.

Our initial screen and follow-up assays represent an important proof of principle, but several questions remain to be addressed. In particular, although our top ‘hit’ ketoconazole markedly affected DLK localization in our primary screen, how this compound acts to reduce DLK palmitoylation and signaling remains to be determined. Nonetheless, findings from some of our additional experiments can help rule out certain possible explanations as to ketoconazole’s mechanism of action.

For example, ketoconazole reduces palmitoylation of DLK and, to a lesser extent, PSD-95, but does not affect palmitoylation of GAP-43 (Fig. 4). These findings stand in contrast to the broad spectrum palmitoylation inhibitor 2BP, suggesting that ketoconazole and 2BP act *via* different mechanisms.

Our results also provide insights into ketoconazole’s mechanism of action. For example, ketoconazole reduces palmitoylation of DLK, which is palmitoylated at a single, internal site, and of PSD-95, which undergoes N-terminal dual palmitoylation. It thus seems unlikely that ketoconazole’s action is defined by the number or location of palmitoylation sites in a given palmitoyl-protein. We speculate that ketoconazole may instead inhibit one or more PAT(s) that can palmitoylate DLK and PSD-95. However, both PSD-95 and DLK can be palmitoylated by a number of different PATs in transfected cells^11,27^, and HEK293T cells express all 23 human PATs^28,29^, so testing this possibility is far from trivial.

Additional insight into ketoconazole’s mechanism of action stems from the initial observation that western blots of HEK293T cell lysates reveal a close doublet of DLK-GFP (Fig. 3C). A plausible explanation for the mobility difference of the two forms of DLK is differential phosphorylation, which is known to affect DLK mobility on SDS-PAGE^30^. Interestingly, both bands of the DLK-GFP doublet are detectable in ABE fractions, suggesting both can be palmitoylated, but the lower band is less prominent in ABE fractions (Fig. 3C). This finding raises the possibility that DLK palmitoylation may be affected by its phosphorylation state, an interesting area for future study. Importantly, though, both 2BP and ketoconazole reduce palmitoylation of both forms of DLK-GFP, suggesting that the action of both of these compounds is independent of DLK phosphorylation state.

It is also informative to consider prior descriptions of ketoconazole’s activity in other contexts. Ketoconazole is an antifungal that was first identified as an inhibitor of enzymes involved in generating ergosterol, the fungal form of cholesterol^31^. In humans, ketoconazole inhibits testicular androgen production and can inhibit the 17α-hydroxylase and 17,20-lyase activities of the steroidogenic P450 enzyme Cytochrome P450 17 A1 (CYP17A1^32^). However, whether these activities relate to ketoconazole’s effects on DLK palmitoylation and signaling is unclear.

The chemical moiety within ketoconazole that acts to reduce palmitoylation is also not fully clear. Interestingly, a second azole-containing compound, sulconazole, was also identified in our screen (Table 1). However, miconazole, a third azole-containing compound that is included in the Prestwick library, was not identified in the screen and was ineffective at reducing DLK puncta at the same concentration as ketoconazole and was toxic at higher concentrations in follow-up assays (D.D.O.M., unpublished observations). Further cheminformatic analysis may facilitate identification of possible common functional moieties present in ketoconazole and/or other screen hits.

While our assay is designed to identify compounds that prevent DLK palmitoylation, it can also be used to identify compounds that reduce DLK stability. Indeed, ketoconazole’s ability to reduce numbers of DLK puncta in our initial screen may in part be due to this secondary activity, because at 10 μM (the concentration used in the initial screen) ketoconazole did slightly reduce total protein expression of DLK-GFP. However, this additional capability of the screening platform is actually an unexpected bonus - given DLK’s role as a key controller of neurodegeneration, compounds that act to destabilize DLK and/or increase DLK degradation might also be of considerable therapeutic benefit. The tight control of DLK levels by ubiquitin-dependent degradation^30,33,34^ suggests that our screen could also identify activators of DLK ubiquitylation and/or inhibitors of DLK de-ubiquitylation. Compounds that act in either of these ways would also be of considerable interest therapeutically.

The reduction in TD-induced c-Jun phosphorylation observed in DRG sensory neurons pre-treated with ketoconazole (Fig. 5) is consistent with this compound acting to reduce DLK palmitoylation and signaling. Indeed, 2BP dramatically reduces TD-induced c-Jun phosphorylation in the same assay (Fig. 5), suggesting that neuronal DLK-JNK-cJun pathway signaling is highly palmitoylation-dependent. Nonetheless, we recognize that effects of ketoconazole on c-Jun phosphorylation may be due to actions of this compound on palmitoylation of targets other than DLK and/or due to non-specific effects. More investigation into the effects of ketoconazole on neuronal signaling is thus warranted.

Finally, dramatic palmitoylation-dependent changes in protein subcellular localization are well described not just for DLK but key regulators of axon integrity, synaptic transmission/higher brain function, and cell growth/proliferation^23,35–40^. Fluorescent- or other epitope-tagged versions of many of these proteins are either readily available or can be easily generated, making HCS a powerful approach to identify small molecules that could affect their localization and activity. While we focused on palmitoylation-dependent changes in the number of DLK puncta per cell, other aspects of protein subcellular localization that can be controlled by palmitoylation (e.g. plasma membrane targeting) can also be quantified by ImageXpress, or by similar software^41^. Our screening platform could thus be readily adapted to identify compounds that affect the palmitoylation-dependent targeting of a variety of therapeutically important proteins (e.g. Ras, oncogenic Src family kinases^40,42,43^) to other subcellular locations. In addition, HCS is also readily compatible with genome-wide RNAi or CRISPR-based screening^17,44^, so our screening platform could be combined with these methods to identify upstream regulators (e.g. PATs and/or thioesterases, or specific binding partners) that control palmitoyl-protein subcellular localization. Such approaches have considerable potential both to provide new biological insights into the control of protein palmitoylation, and also to identify compounds and therapeutic targets to lessen the impact of numerous pathological conditions.

## Materials and Methods

The following antibodies, from the indicated sources, were used in this study: Rabbit anti-GFP (Invitrogen/Thermo Fisher Biosciences: catalog #A11122); mouse anti-GM130 (BD Biosciences, Catalog #610822); Rabbit anti-phospho c-Jun Ser-73 (Cell Signaling Technology, Catalog #3270); DLK/MAP3K12 (Sigma/Prestige, Catalog #HPA039936); mouse anti-PSD-95 (Antibodies Inc., Catalog #75-028); Myc 9E10 (University of Pennsylvania Cell Center, Catalog #3207), rabbit anti-GAPDH (Thermo Scientific, Catalog #PA1-987), mouse anti-tubulin (Millipore Sigma, Catalog #T7451), sheep anti-NGF (CedarLane, catalog #CLMCNET-031).

Wild type DLK-GFP and palmitoyl mutant (C127S) DLK were previously described^11^. pmCherry-NLS was a gift from Martin Offterdinger (Addgene Plasmid #39319)^20^. Rat GAP43 cDNA was gene synthesized (Genewiz) and subcloned into the vector FEW^11^ upstream of a C-terminal myc tag. Untagged PSD-95 cDNA was a gift from Dr. R.L. Huganir (Johns Hopkins University Medical School)^45^. 2-Bromopalmitate (2BP) and S-Methyl methanethiosulfonate (MMTS) were from Sigma. The Prestwick Chemical library was purchased by Temple University’s Moulder Center for Drug Discovery and formulated as 10 mM stocks in DMSO. Fresh Ketoconazole stock for follow-up assays was from LKT Laboratories (Catalog #K1676). All other chemicals were from Fisher Biosciences and were of the highest reagent grade.

### Cell transfection

HEK293T cells were transfected using a calcium phosphate-based method as described previously^36^.

### Transfection and fixation of cells for microscopy

In initial experiments of DLK-GFP subcellular localization, HEK293T cells seeded on poly-lysine-coated coverslips (Warner Instruments) in 6 cm dishes were transfected as above. Cells were treated with 100 µM 2BP 5 h later and then fixed 8 h post-transfection in 4% (wt/vol) paraformaldehyde, 4% (wt/vol) sucrose in PBS. After PBS washes, cells were permeabilized with PBS containing 0.25% (wt/vol) Triton X-100, blocked in 10% (vol/vol) normal goat serum (NGS) in PBS and incubated overnight at 4 °C with rabbit anti-GFP and mouse anti-GM130 antibodies in 10% (vol/vol) NGS, followed by incubation with AlexaFluor-conjugated secondary antibodies for 1 h at room temperature. Nuclei were stained with 300 nM DAPI in PBS for 10 min and coverslips were mounted in Fluoromount G (Southern Biotech) before imaging.

For High Content Screening assays, HEK293T cells were plated in poly-lysine coated 96 well plates (Greiner Bio-One, black walled chimney-wells), transfected as above and treated with 2BP (10 μM final concentration), library compounds or DMSO vehicle control at 2 h post-transfection. The Prestwick Compound Library was spotted onto 96 well plates at 10 mM in DMSO and resuspended in 200 µL pre-warmed DMEM. 40 µL of diluted drug was then added to cells in 160 µL of DMEM (containing glutamax, 10% FBS and antibiotics) in duplicate. Cells were incubated with compounds at 37 °C for a further 14 h. Subsequently, medium was removed and cells were fixed in 4% PFA (1x PBS) for 20 mins at RT, washed twice with PBS and stained with 300 nM DAPI for 30 mins at RT, followed by 2 washes of PBS.

### High Content Screening

High Content screening was performed using the ImageXpress micro high content imaging system (Molecular Devices, Downingtown, PA) driven by MetaXpress software. Six images per well were acquired in each of three channels (DAPI, FITC, TRITC) at 10X magnification in an unbiased fashion. Images were analyzed using the MetaXpress ‘Multiwavelength Scoring’ (for mCherry-NLS signals) and ‘Transfluor’ modules (for DLK-GFP signals). Data were exported to a spreadsheet using the AcuityXpress software package (Molecular Devices).

### Thresholding

Compounds that reduced either DAPI or NLS signals by greater than 30% of the average of vehicle-treated controls for each day were excluded from analysis due to likely cytotoxicity and/or broad effects on transcription, translation or protein stability. MetaXpress imaging software was then used to determine the effect of the remaining compounds on DLK puncta (“Total Puncta Count” option, from DLK-GFP signal) and total number of transfected cells (from mCherry-NLS signal). The term “DLK puncta per transfected cell” was used for this readout because punctate DLK-GFP distribution is likely a mixture of Golgi-associated and vesicle-associated pools of DLK. Compounds that reduced DLK puncta per transfected cell by 3 times the standard deviation of the mean of all vehicle-treated controls were considered “Hits”.

### Follow-up Dose-dependence Assay

To confirm the effect of ketoconazole, HEK293T cells were seeded on poly-lysine-coated 96 well plates and transfected as for the primary assay with DLK-GFP and mCherry-NLS cDNAs. Two hours post-transfection, cells were treated with freshly dissolved ketoconazole over a 5-point dilution range, or with DMSO vehicle control. Cells were fixed 16 h later and processed and imaged as for the primary screen.

### Follow-up Golgi Colocalization Assay

To determine the effect of ketoconazole on DLK-GFP targeting to the Golgi, HEK293T cells were seeded on poly-lysine-coated coverslips and transfected with wtDLK-GFP cDNA as above. Cells were treated with 10 µM ketoconazole or DMSO vehicle 2 h later. Sixteen hours after ketoconazole treatment, cells were fixed and then immunostained with anti-GM130 and anti-GFP antibodies, followed by incubation with AlexaFluor568- and AlexaFluor647-coupled secondary antibodies. Confocal images of transfected cells were acquired using a Leica SP8 confocal microscope (40x, 1.2NA oil immersion objective). Maximum intensity projections of z-stack images were generated and saved as TIFF files, which were then opened in Fiji. GM130 images of each sample were thresholded to an identical value in Fiji to create a mask of the Golgi apparatus within a given cell. A Region of Interest (ROI) corresponding to the total DLK-GFP signal from that same cell was then manually drawn in Fiji. The Total Integrated Intensity of the DLK-GFP signal was logged to a spreadsheet. The ‘Analyze Particles’ function was then used to quantify the fraction of the DLK-GFP signal that overlapped with the GM130 mask. The ratio of GM130-overlapping DLK-GFP signal: total DLK-GFP signal was then calculated and plotted.

### Cytotoxicity Assay

HEK293T cells were seeded on poly-lysine-coated dishes and transfected with wtDLK-GFP cDNA as above. Cells were treated with ketoconazole over a five-point dilution range 2 h later. Sixteen hours after ketoconazole treatment, cells were washed twice with room temperature PBS and then incubated with 1 μg/ml Propidium Iodide (PI) in PBS for 30 min at 4 °C. Cells were then washed twice more with PBS and live images of DLK-GFP fluorescence and PI staining were acquired. Images were manually counted to assess the total number of transfected cells (without thresholding) and PI-positive cells per field.

### Palmitoylation assay

Palmitoylation of transfected proteins in HEK293T cells was assessed by acyl biotin exchange assays, as previously described^36^ except that cells were cultured in 6 well plates and bands were imaged and quantified using a LiCOR Odyssey system. Images were prepared and analyzed using Image Studio Lite Ver 4.0.

### NGF Withdrawal

Primary dorsal root ganglion (DRG) neurons were prepared from embryonic day 15.5 rat embryos, as previously described^11^. All procedures followed National Institutes of Health guidelines and were approved by the Institutional Animal Care and Use Committee (IACUC) of Temple University. At 7 days *in vitro* DRG neurons were pretreated with 2.5 µM Ketoconazole overnight or 20 µM 2BP for 2 h prior to withdrawal of NGF in the presence of sheep anti-NGF IgG in the continued presence of drug. Cells were then lysed in SDS-PAGE loading buffer and processed for subsequent SDS-PAGE and subsequent immunoblotting. Images were acquired and analyzed as above.

### Statistical analysis

Where indicated, the non-parametric one-way ANOVA Kruskal-Wallis test was performed with a Dunn’s multiple comparison *post-hoc* analysis. In addition, 2-way ANOVA was performed with Bonferroni *post-hoc* analysis. All error bars represent SEM.

### Ethical approval

All procedures involving experimental animals followed National Institutes of Health guidelines and were approved by the Institutional Animal Care and Use Committee (IACUC) of Temple University.

## Supplementary information

Martin et al All Supplementary Information

## Data Availability

The datasets generated during and/or analyzed during the current study are available from the corresponding author upon reasonable request.
