# Supplementary material for: Identification of Novel Inhibitors of DLK Palmitoylation and Signaling by High Content Screening
Source: Sci Rep. 2019 Mar 6;9:3632. doi: 10.1038/s41598-019-39968-8 (PMC6403299; doi:10.1038/s41598-019-39968-8)

**Supplementary Information for "Identification of Novel Inhibitors of DLK Palmitoylation and Signaling by High Content Screening"**

Dale D.O. Martin, Prasad S. Kanuparthi, Sabrina M. Holland, Shaun S. Sanders, Hey-Kyeong Jeong, Margret B. Einarson, Marlene A. Jacobson and Gareth M. Thomas

**Supplementary Figure 1. Multiple readouts of DLK punctate distribution are highly palmitoylation-dependent in HEK293T cells.** Images of DLK-GFP expressing HEK293T cells were analyzed using the 'Transfluor' modules within MetaXpress analysis software to quantify changes in different aspects of punctate DLK signals, in particular "Puncta Count," "Puncta Count Per Cell," "Puncta Total Area," "Puncta Area Per Cell," "Puncta Integrated Intensity," and "Puncta Average Intensity", as indicated. Error bars represent SD. Z-prime value (z′) for each metric is indicated. All measurements reached a significance of 0.0001 by unpaired parametric two-tailed t-test.

**Supplementary Figure 2. Concentrations of ketoconazole used in High Content Screening are not Significantly Cytotoxic in HEK293T cells. A)** HEK293T cells in 6cm dishes were transfected with DLK-GFP and treated with the indicated concentrations of ketoconazole prior to incubation with Propidium Iodide. DLK-GFP–positive cells (without threshold; gray trace, right hand axis) and the fraction of DLK-GFP–positive cells that stained positive for Propidium Iodide (red trace, left hand axis) per field were quantified manually from live images. Ketoconazole did not significantly increase the fraction of Propidium Iodide-positive cells at any concentration tested (ANOVA with *post hoc* Bonferroni test). Data are from 4 fields per well from n=3 wells per condition.

Error bars indicate SEM. **B)** The total number of intact nuclei per field (assessed using DAPI signals) was quantified for HEK293T cells in 96 well plates transfected with DLK-GFP and subsequently treated with the indicated concentrations of ketoconazole. Samples used to generate these data were also used for Figure 3A and DAPI counts plotted here were extracted from MetaXpress analysis.*; $p=0.0025$, $F(6)=3.775$ versus vehicle-treated control, ANOVA with *post hoc* Bonferroni correction, n=8 determinations per condition.

**Supplementary Figure 3. Full-length blots of those cropped and presented in the main Figures.**

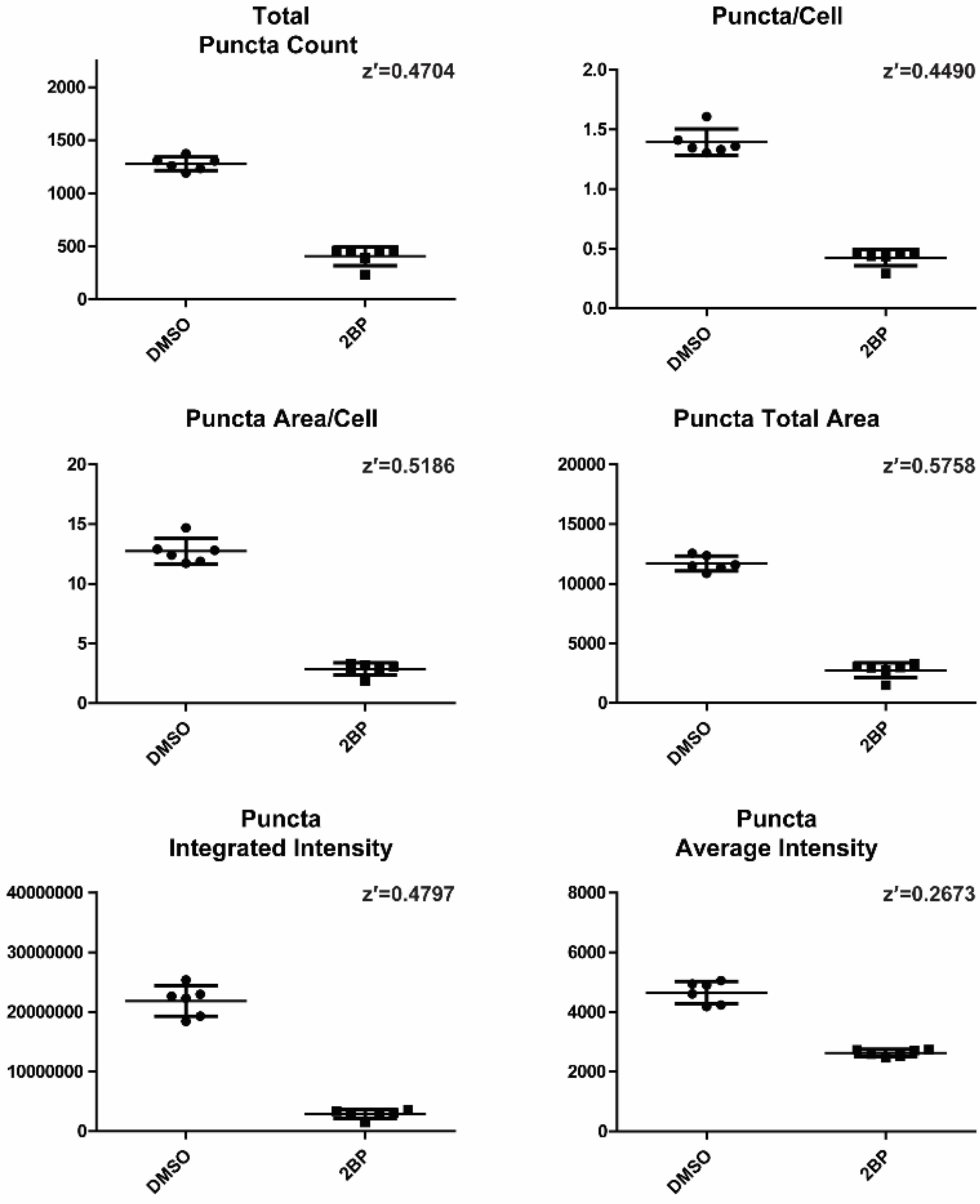


Martin *et al.*, Supplemental Figure 1

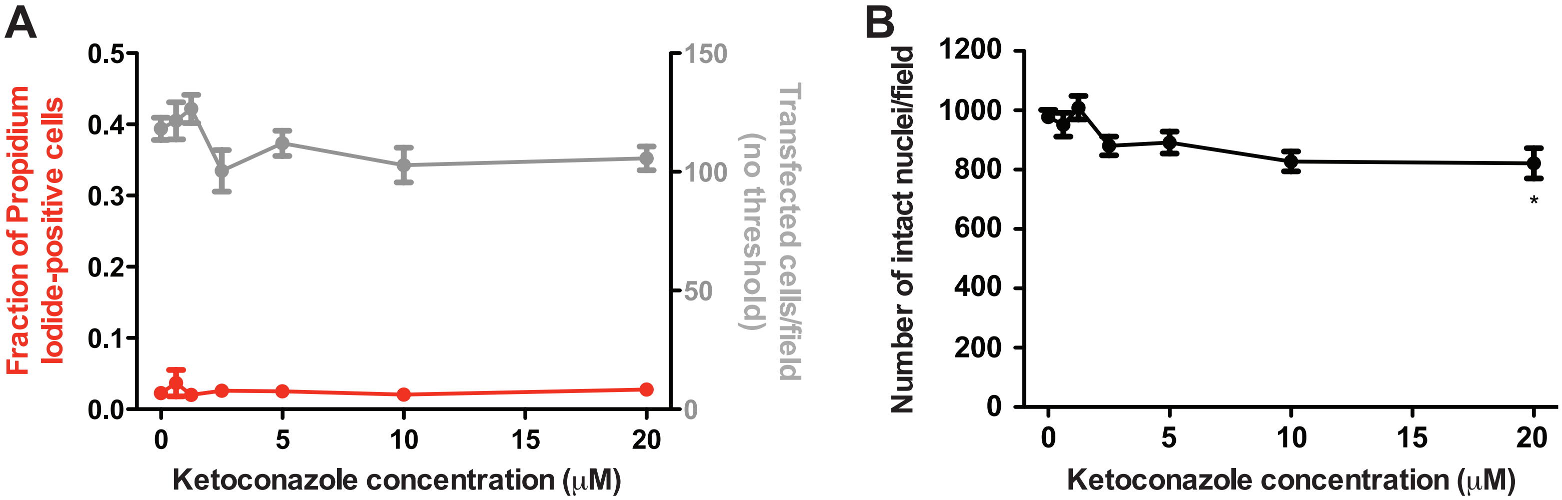


Martin et al. Supplemental Figure 2.

## Figure 3C

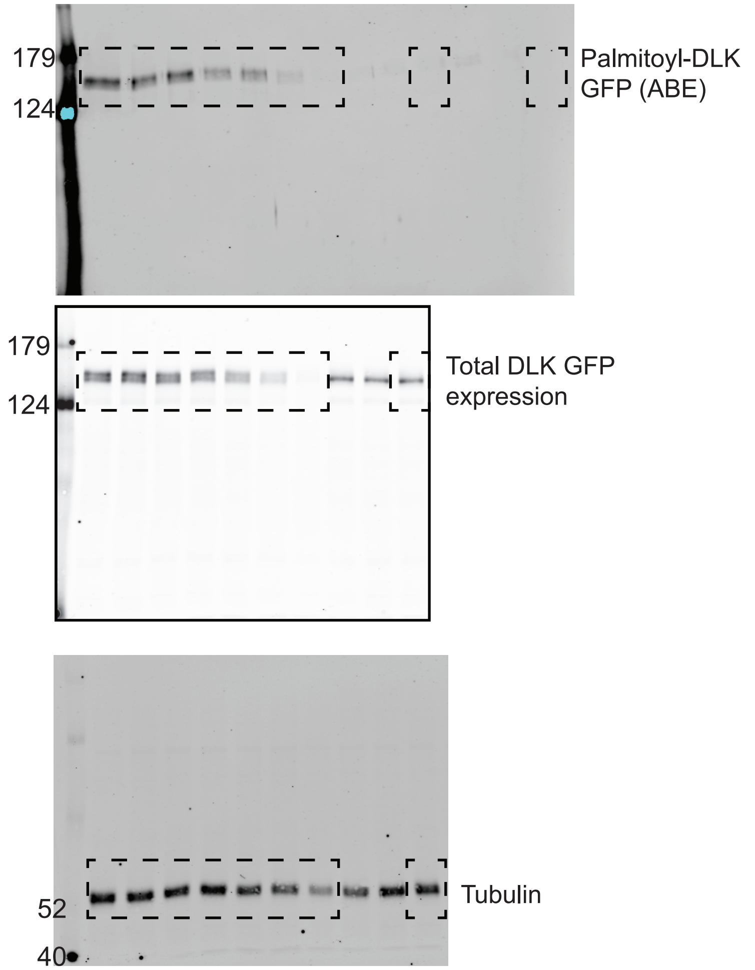


## Figure 5A

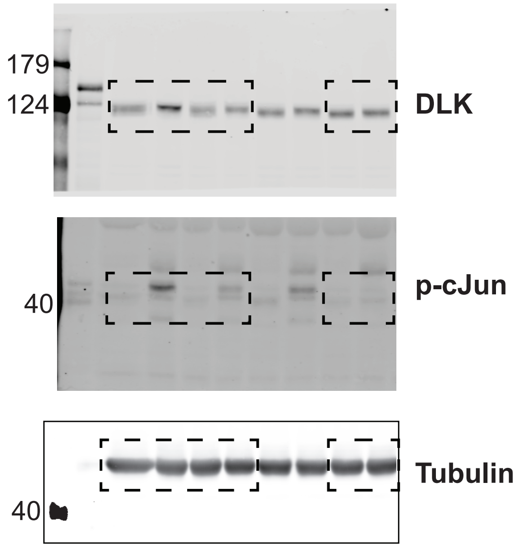


## Figure 4A

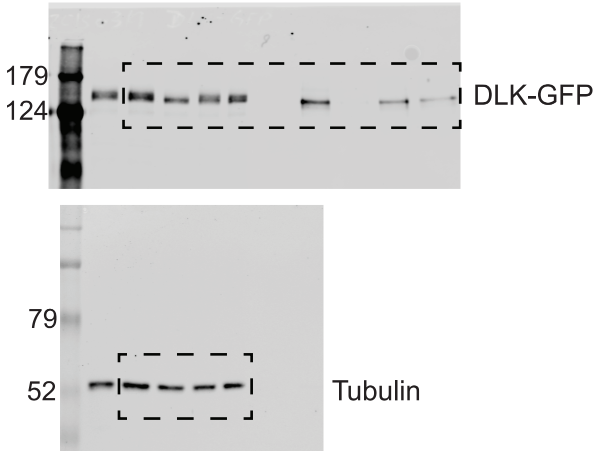


## Figure 4C

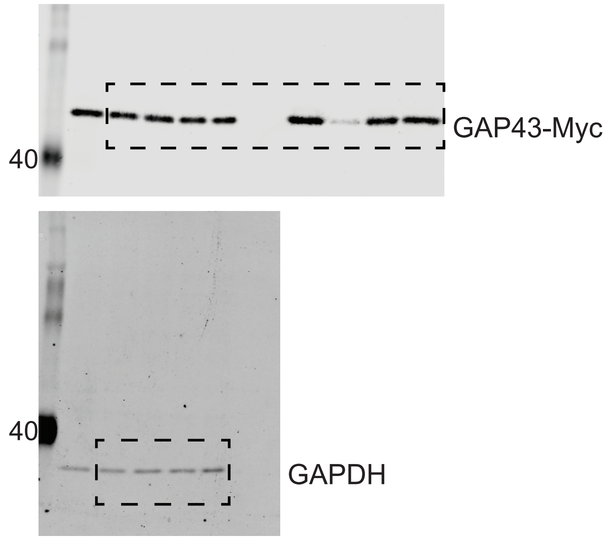


## Figure 4E

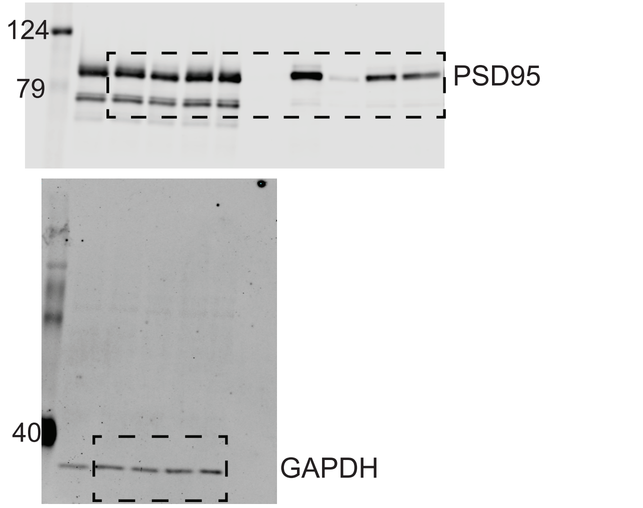

Supplement: Supplementary file 1 — Martin et al All Supplementary Information [file 41598_2019_39968_MOESM1_ESM.pdf]
